# SIRT5 prevents mitochondrial dysfunction and cardiac hypertrophy induced by RIP140

**DOI:** 10.22038/ijbms.2024.80343.17390

**Published:** 2025

**Authors:** Liying Liang, Yi Huang, Qiujuan Wang, Ye Hong, Honghui Zhen, Yanfang Chen

**Affiliations:** 1 Department of Pharmacy, Guangzhou Eighth People’s Hospital, Guangzhou Medical University, Guangzhou, Guangdong, China; 2 Guangzhou Special Service Recuperation Center of PLA Rocket Force, Guangzhou, Guangdong, China; # These authors contributed equally to this work

**Keywords:** Cardiac hypertrophy, Energy metabolism Mitochondria, RIP140, SIRT5

## Abstract

**Objective(s):**

To investigate the effect and mechanism of sirtuin5 (SIRT5) on mitochondrial dysfunction and cardiac hypertrophy induced by receptor-interacting protein 140 (RIP140).

**Materials and Methods:**

The neonatal rat cardiomyocytes (NRCMs) and SD rats were treated with Angiotensin II (Ang II) to induce *in vitro* and *in vivo* model of cardiac hypertrophy. RIP140 was overexpressed by adenovirus infection, and SIRT5 was overexpressed by plasmid transfection. RIP140 and SIRT5 were knocked down by siRNA interference. The expression of RIP140, SIRT5, and biomarkers of cardiac hypertrophy were measured by qRT-PCR and western blot. The transcription levels of mitochondrial DNA-encoded genes were detected by qRT-PCR. Cell surface area and mitochondrial membrane potential were respectively detected by rhodamine-phalloidin and tetramethylrhodamine ethyl ester (TMRE) fluorescence analysis. Cellular oxygen consumption and ATP production were investigated using assay kits. All data are from at least three independent experiments.

**Results:**

The expression of SIRT5 was down-regulated in NRCMs and hearts treated with Ang II. Overexpression of SIRT5 protected cardiomyocytes from AngII-induced hypertrophy, whereas knockdown of SIRT5 resulted in cardiac hypertrophy. Moreover, since SIRT5 was regulated by the transcriptional coactivator, we also found that SIRT5 could be negatively regulated by the transcriptional corepressor RIP140 in cardiomyocytes. Furthermore, SIRT5 significantly attenuated energy metabolic dysregulation and mitochondrial dysfunction and exerted its protective role on myocardial hypertrophy under the regulation of RIP140.

**Conclusion:**

SIRT5 exerts a protective role in mitochondrial dysfunction and cardiac hypertrophy induced by RIP140.

## Introduction

Cardiac hypertrophy is the heart’s compensatory response to various physiological or pathological stimuli, characterized by increased protein synthesis, cell size, and reactivation of the fetal gene program at the cellular level (1). However, prolonged hypertrophy ultimately progresses to deterioration in heart function and eventually results in heart failure or sudden death. The incidence of cardiac hypertrophy increases dramatically with age, implying that aging-associated mechanisms may be crucial for the molecular regulation of cardiac hypertrophy (2).

As a highly energy-consuming organ, the heart requires a continuous supply of Adenosine Triphosphate (ATP) synthesis to maintain its contractile function. Most ATP is produced by the mitochondria in eukaryotic cells through fatty acid oxidation and oxidative phosphorylation (3). It was hypothesized that impaired substrate metabolism contributed to cardiac remodeling and contractile dysfunction, which accelerated heart failure in response to several kinds of pathological stimuli (4, 5). Therefore, targeting myocardial mitochondrial metabolism might be a potential therapy to prevent heart failure progression. 

Sirtuins are a family of evolutionarily conserved histone deacetylases (HDACs) that play pivotal roles in dynamically regulating the maintenance of metabolic homeostasis (6, 7). Among the seven sirtuins, SIRT1-7 and SIRT3-5 are located in the mitochondria and are mainly involved in regulating cellular mitochondrial metabolism (6, 8). Our previous studies demonstrated that SIRT3 knockdown increased the acetylation level of NMNAT3 and subsequently lowered mitochondrial NAD levels, resulting in cardiac hypertrophy (9). Apart from SIRT3, SIRT5 has also been reported to have important regulatory roles in energy metabolism and mitochondrial function (10, 11). SIRT5, mainly expressed in the heart, brain, and other highly energy-consuming organs, has been discovered as the only protein with desuccinase activity at present since protein lysine succinylation was found to predominantly accumulate in the heart of SIRT5 KO mice (12, 13). More importantly, SIRT5 could protect against oxidative stress-induced cardiomyocyte apoptosis (14), while knockout of SIRT5 could exacerbate ischemia/reperfusion injury (15). However, the expression of SIRT5 in cardiovascular diseases such as myocardial hypertrophy and its regulatory mechanism related to cardiac energy metabolism are poorly defined.

Collective evidence indicates that the metabolic gene activation or repression depends on recruiting a coactivator or corepressor. As a key upstream regulator of energy expenditure, Receptor-interacting protein 140 (RIP140) has been reported to suppress the expression of gene clusters involved in substrate metabolism (16, 17). Overexpression of RIP140 could impair mitochondrial structure and biogenesis in hearts and despair mitochondrial oxygen consumption and ATP synthesis during the progression of heart failure after LAD surgery (18-20). Coincidently, it was also reported that RIP140 deficiency enhanced cardiac fuel metabolism and protected mice from heart failure (21). Therefore, these observations suggest that RIP140 can induce disturbances in myocardial energy metabolism and thus exacerbate the progression of cardiac disease.

It is well known that transcriptional corepressor RIP140 and coactivator PPARc coactivator-1α (PGC-1α) are opposing-functional regulators in maintaining energy balance. Recent research revealed that SIRT5 was regulated by PGC-1α, which shared many common downstream targets with RIP140 (22). Our previous studies uncovered that exogenous overexpression of RIP140 resulted in myocardial hypertrophy and cardiac dysfunction by inhibiting mitochondrial function (18, 19). Hence, whether RIP140 could regulate SIRT5 in cardiomyocytes and their regulatory mechanism in developing cardiac hypertrophy is worthy of further investigation. In this study, we verified that SIRT5 exerted a protective effect on Ang II-induced hypertrophy. Furthermore, RIP140 repressed SIRT5, and the inhibition of SIRT5 participated in RIP140-induced mitochondrial dysfunction and cardiac hypertrophy. These findings highlight SIRT5 as a novel target of RIP140, governing cardiac mitochondrial metabolism during the progression of cardiac hypertrophy.

## Materials and Methods

### Materials

Dulbecco’s modified Eagle’s medium (DMEM) and fetal bovine serum (FBS) were obtained from Gibco. 4’, 6-diamidino-2-phenylindole (DAPI), penicillin/streptomycin, and Lipofectamine 2000 were purchased from Life Technologies/ Invitrogen. Anti-SIRT5 and anti-RIP140 antibodies were purchased from Abcam. Anti-BNP and anti-β-MHC antibodies were obtained from ABclonal. Anti-ANF and anti-α-tubulin antibodies were purchased from Santa Cruz and Sigma-Aldrich, respectively. Ang II was purchased from Sigma-Aldrich. Detailed information of the materials are listed in Table 1.

### Primary culture of neonatal rat cardiomyocytes (NRCMs)

Primary culture of neonatal rat cardiomyocytes (NRCMs) was prepared using a validated method. Briefly, Cells from the hearts of 1- to 3-day-old Sprague-Dawley (SD) rats were seeded at a density of 1×10^6^ cells/well onto 6-well plates in DMEM supplemented with 10% fetal bovine serum (FBS) and 5-bromodeoxyuridine (0.1 mM) and then cultured in DMEM with 10% fetal bovine serum and 0.1 mM bromodeoxyuridine at 37 ^°^C in 5% CO_2_ humidified air as described. All experimental procedures complied with the Guide for the Care and Use of Laboratory Animals published by the US National Institutes of Health (NIH Publication No. 85-23, revised 1996) and were approved by the Institutional Ethics Review Board of Sun Yat-Sen University.

### Animal models of cardiac hypertrophy and experimental protocols

Male Sprague-Dawley rats weighing 220 g to 250 g were purchased from the Animal Breeding Center of Sun Yat-sen University. The number of rats was 5 per group. Ang II (2 mg/kg/d, Sigma-Aldrich) dissolved in saline was injected subcutaneously without anesthesia for two weeks to induce an animal model of cardiac hypertrophy. Control animals underwent the same procedure, except that saline vehicle was injected subcutaneously. On the last day of treatment, two-dimensionally guided M-mode echocardiography was performed with a Technos MPX ultrasound system (ESAOTE, Italy) to confirm typical concentric hypertrophy of the rat heart, according to our previous report (23). Subsequently, the rats were anesthetized by intraperitoneal injection of 0.45% pentobarbital sodium (45 mg/kg), and the blood was sampled from the abdominal aorta. After 0.1 mol/L KCl injection to stop the heart beating during diastole, the rat hearts were rapidly removed, weighed, and then quickly frozen in liquid nitrogen and stored at -80 ^°^C for subsequent experiments. The Ethics Committees of Sun Yat-sen University of Medical Science approved the experiments.

### RNA interference

Small interference RNA (siRNA) for SIRT5 (siSIRT5) and negative control siRNA were purchased from Genepharma (Shanghai, China). Cardiomyocytes seeded in 35 mm^2^ dishes were transfected with 100 pmol of the targeted or negative control siRNA, respectively, using 5 μl lipofectamine 2000 (Invitrogen) according to the manufacturer’s instructions. The qRT-PCR was performed to compare the silencing efficiency of different siRNAs. 

The oligo sequences used for RNA interference were as follows: 

SIRT5: Sense 5’-CCAACAGAUUCAGGUUUCATT-3’, 

Antisense 5’- UGAAACCUGAAUCUGUUGGTT-3’; 

RIP140: Sense 5’-GCCGUAGAUAAUGCCAAUATT-3’,

Antisense 5’- UAUUGGCAUUAUCUACGGCTT-3’;

Negative control: Sense 5’-UUCUCCGAACGUGUCACGUTT-3’, 

Antisense 5’- ACGUGACACGUUCGGAGAATT-3’

### Plasmid and transfection

The SIRT5-Flag (SIRT5) plasmid was purchased from Add Gene. The plasmid encodes the rat full-length sequence of SIRT5. Cardiomyocytes were transiently transfected with 4 μg of SIRT5 or empty vector using Lipofectamine 2000 (Invitrogen) according to the manufacturer’s instructions. Next, the cells were cultured in a complete medium for 24 hours, followed by further treatments and assays.

### RNA isolation and quantitative RT-PCR (qRT-PCR)

The total RNA of the cultured cells was extracted using TRI-zol reagent (Invitrogen, Carlsbad, CA, USA) following the manufacturer’s instructions. According to the instructions, one microgram of total RNA was reversely transcribed to cDNA using a reverse transcription Kit (Thermo Scientific, Waltham, MA, USA). Real-time quantitative PCR was performed with the above-prepared cDNA and SYBR Green Master Mix (Thermo Scientific, Waltham, MA, USA) in a real-time PCR amplifier (Thermo Scientific, Waltham, MA, USA). The amplification conditions were 7 min at 95 ^°^C, followed by 40 cycles of 10 sec at 95 ^°^C, 30 sec at 60 ^°^C and 10 sec at 20 ^°^C. A dissociation curve was generated to verify that the majority of fluorescence detected could be attributed to the labeling of specific PCR products and to verify the absence of primer-dimers and sample contamination. Primers designed for amplification were synthesized by Sangon Biotech Co Ltd (Shanghai, China), and the housekeeping gene β-actin was used to normalize the mRNA level of each sample. Data were shown as fold change over the control group. 

The primers used in this study were as follows in Table 2. 

### Western blot

Experiments were performed as described previously. Protein extracts from cells were separated by electrophoresis in SDS-PAGE gel, transferred to PVDF membranes (Millipore), and incubated with primary antibodies, followed by appropriate horseradish peroxidase (HRP)-conjugated secondary antibodies. Bands were developed with a super signal chemical luminescent substrate (Thermo) and visualized by the LAS4000 imager (GE Healthcare, Waukesha, WI, USA). The intensities of the blots were quantified by ImageQuantTL (GE Healthcare) software, and the intensity of each protein band was normalized by that of α-tubulin. Detailed information on the antibodies is listed in Table 3 .

### Oxygen consumption assay and intracellular ATP levels

NRCMs were cultured in a 96-well clear bottom plate. Measurements were performed in fresh DMEM containing 6.25% MitoXpress ® Xtra reagent (Luxcel Biosciences) covered with pre-warmed Mineral Oil using a fluorescence plate reader-FLUOstar Omega BMG Labtech, Germany, kinetically for 150 min at 37 ^°^C. Excitation and emission wavelengths were 380 and 650 nm, respectively. Oxygen consumption was determined by the average slope of the relative fluoresce unit (RFU) as recommended. ATP levels normalized by protein concentrations in NRCMs were measured with the ATP Assay Kit (Beyotime, China) according to the manufacturers’ recommendations.

### Measurement of cell surface area

Cardiomyocytes seeded on 6-well plates were fixed with 4% paraformaldehyde for 20 min at room temperature, followed by 1% Triton-100 treatment for 10 min. After incubation with 0.1% rhodamine-phalloidin for 30 min, the cells were washed with PBS and further incubated with DAPI. The images of cardiomyocytes were detected by a confocal microscope (Zeiss 710), and the cell surface area from randomly selected fields (50 for each group) was determined by the built-in image analysis software.

### Recombinant adenovirus construction

The adenovirus encoding RIP140 (Ad-RIP140) was constructed using the AdEasy transfer vector, as previously described (19). The full-length RIP140 gene was cloned into the pAdTrack CMV shuttle vector and then recombined with the viral skeleton pAdEasy-1 vector in BJ5183 bacteria. Null control virus (Ad-Null) was prepared by recombining the pAdTrack CMV and pAdEasy-1 vectors. Both Ad-RIP140 and Ad-Null harboring GFP markers were driven by cytomegalovirus promoters. After recombination, adenovirus was propagated and amplified in the AD-293 cell line. The identified adenovirus was purified by filtration, according to Adeno-X Maxi purification kit (Clontech) instructions, then diluted to a suitable titer before use. 

### Statistical analysis

Data were presented as means±standard error of the mean (SEM). Statistical analysis was performed using SPSS statistic software13.0. The mean difference between the two groups was tested using the Student’s t-test. Statistical analysis among the various groups was performed using a one-way analysis of variance (ANOVA) with Bonferroni *post hoc* test. In all cases, the difference between groups was considered statistically significant at *P*<0.05. All results were from at least three independent experiments.

## Results

The expression of SIRT5 was down-regulated in neonatal rat cardiomyocytes (NRCMs) and hearts were treated with Ang II. 

**Figure 1:**
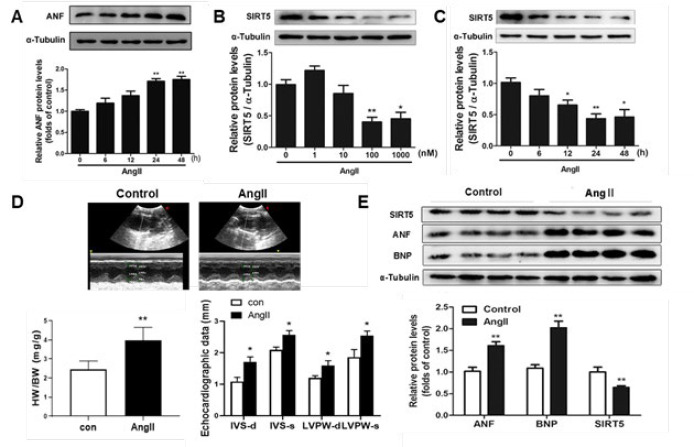
The protein expression of SIRT5 was decreased in NRCMs and hearts of rat treated with Ang II

Ang II is a well-established neurohumoral factor that can stimulate cardiac hypertrophy. As shown in Figure 1A, Ang Ⅱ treatment (100 nM) caused a significant increase of hypertrophic biomarker ANF in NRCMs at 24 hr and 48 hr. To detect the expression changes of SIRT5 in Ang Ⅱ-induced cardiac hypertrophy, NRCMs were treated with AngⅡ in different doses or times indicated. Compared with the control group, the protein expression of SIRT5 declined in NRCMs in a dose- and time-dependent manner responding to Ang II, showing the most significant decrease of 100 nM Ang Ⅱ stimulation at 24 hr (Figure 1B, C). In addition, equal normal saline or Ang II infusion (2 mg/kg/d) for two weeks in SD rats and then echocardiography were conducted to confirm that the *in vivo* model of cardiac hypertrophy was successfully established. As shown in Figure 1D, the results in heart weight/body weight ratio (HW/BW ratio), echocardiographic graph, and parameters, including interventricular septum thickness (IVS) and left ventricular posterior wall (LVPW) in the diastolic and systolic period, were significantly increased in the AngII group compared with the control group. In accordance with results from NRCMs, a remarkable up-regulation of hypertrophic biomarker (ANF and BNP) and down-regulation of SIRT5 was also discovered in AngII-treated hearts (Figure 1E).

### SIRT5 protected cardiomyocytes from Ang II-induced hypertrophy

**Figure 2:**
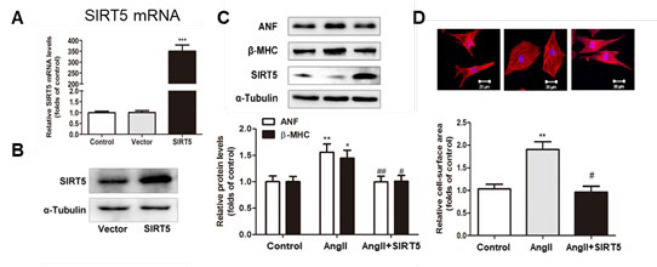
Overexpression of SIRT5 protected rat cardiomyocytes from AngII-induced hypertrophy

**Figure 3:**
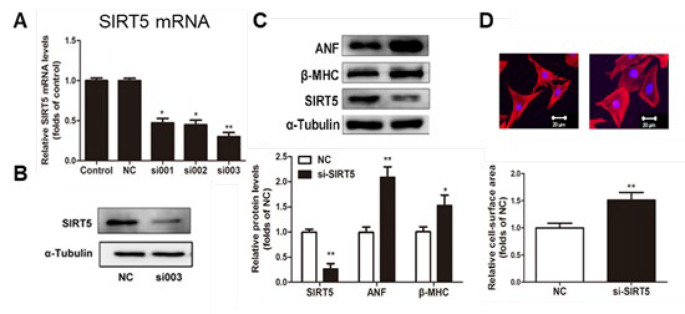
Knockdown of SIRT5 induced cardiac hypertrophy

To investigate the potential effect of SIRT5 on cardiac hypertrophy, NRCMs were transfected with SIRT5 plasmid or siRNA. As shown in Figure 2A and B, both mRNA and protein levels of SIRT5 were significantly enhanced in cardiomyocytes transfected with SIRT5 plasmid. Subsequently, the cells were treated with 100 nM Ang II for 24 hr; the cellular hypertrophy response was indicated by increased cell surface area and activation of fetal gene expression. As shown in Figure 2C and D, overexpression of SIRT5 could significantly attenuate Ang II-induced hypertrophic response. Furthermore, cardiomyocytes were transfected with oligo sequence used for RNA interference of SIRT5 to knock down endogenous SIRT5. Quantitative RT-PCR was performed to evaluate the effect of three independent siRNAs, marked si001, si002, and si003. Among these three siRNAs, si003 reduced the mRNA and protein expression of SIRT5 by 60% (*P*<0.01, compared with control), exhibiting potential efficacy for SIRT5 silence (Figure 3A, B). Therefore, si003, namely siSIRT5, was used in the following experiments. After transfection, the protein levels of ANF and β-MHC and cell surface area were measured. The results revealed that the knockdown of SIRT5 by si-SIRT5 could mimic the effects of RIP140 on cardiac hypertrophy by enhancing the expression of hypertrophic biomarkers and cell surface area (Figure 3C, D). 

### SIRT5 was negatively regulated by RIP140 in cardiomyocytes

**Figure 4:**
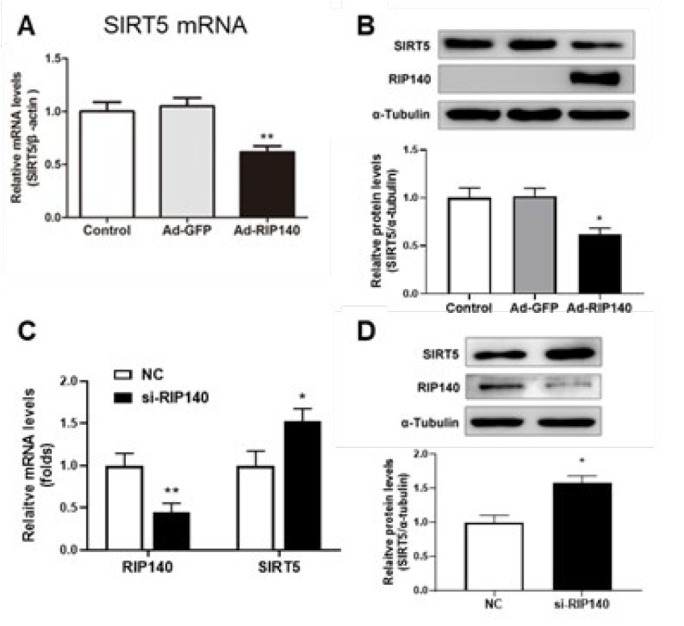
The expression of SIRT5 was changed in primary NRCMs with overexpression or knockdown of RIP140

In contrast to the protective role of SIRT5, RIP140 is widely recognized for its involvement in metabolic and mitochondrial dysfunction, thus promoting cardiac hypertrophy and subsequent transition to heart failure (18-21). Given that SIRT5 is regulated by PGC-1α, a transcriptional coactivator sharing numerous downstream targets with the transcriptional corepressor RIP140, we next investigated whether RIP140 regulates SIRT5 on myocardial hypertrophy. The mRNA and protein expression of SIRT5 were examined in cardiomyocytes transfected with Ad-RIP140 or si-RIP140 through quantitative RT-PCR and western blot analysis. We found that both mRNA and protein expression of SIRT5 declined in cardiomyocytes overexpressing RIP140, compared with the control and Ad-GFP group (Figure 4A, B). To further verify the inhibitory effect of RIP140 on SIRT5, we detected changes in SIRT5 expression after siRNA interference with RIP140 expression. Conversely, results shown in Figures 4C and D revealed that interference with RIP140 significantly up-regulated the expression of SIRT5. The above results indicated that both the transcriptional and protein levels of SIRT5 were negatively regulated by RIP140. 

### SIRT5 prevented cardiomyocyte hypertrophy induced by RIP140

**Figure 5:**
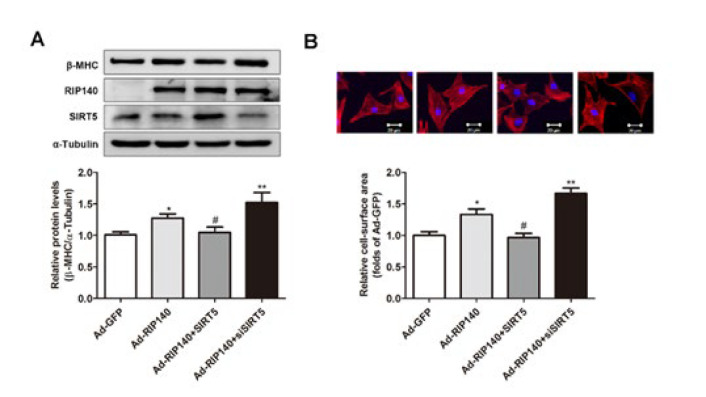
Effect of SIRT5 on RIP140-induced cardiac hypertrophy

In order to confirm the regulation between RIP140, a transcriptional corepressor that plays an important role in energy metabolism, and the mitochondrial protein SIRT5 during the development of cardiac hypertrophy, we next measured the cell surface area and activation of fetal gene expression to evaluate the effect of SIRT5 on RIP140-induced cellular hypertrophy response in NRCMs. As shown in Figure 5A and B, overexpression of SIRT5 could signiﬁcantly attenuate the increase in β-MHC expression and cell surface area induced by RIP140 overexpression. In addition, knockdown of SIRT5 with siSIRT5 could aggravate hypertrophic responses caused by Ad-RIP140 transfection as indicated by further elevation of hypertrophic biomarker and cell-surface area. These observations indicated that SIRT5 exerted its protective role on myocardial hypertrophy under the regulation of RIP140.

### SIRT5 inhibited RIP140-induced energy metabolic dysregulation and mitochondrial dysfunction in NRCMs

**Figure 6:**
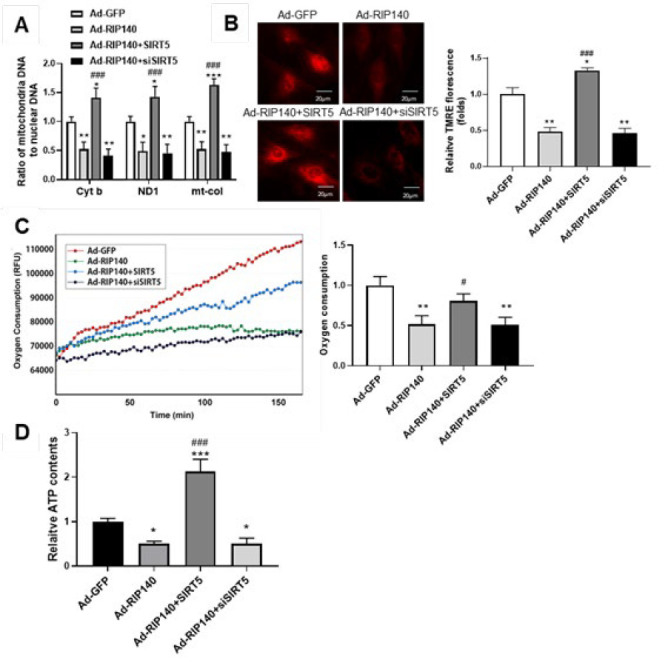
SIRT5 relieved RIP40-induced dysregulation of key metabolic genes expression and mitochondrial dysfunction in NRCMs

As RIP140 has been found to cause impairment of mitochondrial oxidation and cellular metabolism, the impact of SIRT5 on RIP140-induced metabolic gene expression and mitochondrial dysfunction was investigated by co-treatment with Ad-RIP140 and SIRT5-overexpressing plasmid (or siSIRT5). As shown in Figure 6A, mitochondrial DNA-encoded genes, including NADH dehydrogenase subunit 1(ND1), cytochrome b (Cyt b), and mitochondrially encoded cytochrome C oxidase I (mt-co1), were diminished in cardiomyocytes treated with Ad-RIP140, whereas the expression of these genes was completely restored upon transfection with SIRT5. Conversely, knockdown of SIRT5 with siSIRT5 slightly exacerbated the down-regulation of metabolic genes induced by RIP140 overexpression. Moreover, mitochondrial membrane potential detected by TMRE ﬂuorescent dye was significantly decreased in Ad-RIP140 group, which could be recovered by the combined treatment of Ad-RIP140 infection and SIRT5 transfection (Figure 6B). Additionally, cellular oxygen consumption and ATP production were measured to evaluate the capacity of mitochondria in producing energy. As shown in Figure 6C, loss of oxygen consumption in Ad-RIP140-infected cells was remarkably rebounded by transfecting with SIRT5-overexpressing plasmid. In line with the oxygen consumption studies, overexpressing SIRT5 also reversed the decline in ATP production induced by RIP140. Similar to the results observed in metabolic gene expression, we also found that siSIRT5 had a mild exacerbation of RIP140-caused mitochondria dysfunction (Figure 6D).

These observations provided further evidence that SIRT5 overexpression prevented RIP140-induced energy metabolic dysfunction and mitochondrial dysfunction, whereas SIRT5 knockdown aggravated RIP140-induced cardiac hypertrophy and impairment of mitochondrial function.

## Discussion

It is widely recognized that abnormality of energy metabolism results in cardiac dysfunction and even heart failure (2, 24-26). During the development of heart failure, the capacity for fatty acid oxidation (FAO) and ATP production of cardiomyocytes is progressively diminished, contributing to pathologic cardiac hypertrophy and contractile dysfunction. The oxidative metabolism of fatty acids and ATP generation by mitochondria are essential for cardiac contractile function (27-30). In recent years, the mitochondrial sirtuin SIRT5 has been reported to regulate mitochondrial dynamics, ATP generation, and ROS detoxification (13, 31-33). Furthermore, SIRT5 also exhibits multiple protection effects on cardiomyocytes and down-regulation of SIRT5 has been observed in animal models of cardiac dysfunction (12, 14, 15, 34). In line with these findings, our present study demonstrated that the transcriptional and protein levels of SIRT5 were decreased in Ang II- stimulated cardiac hypertrophy* in vivo* and* in vitro* (Figure 1). Additionally, overexpression of SIRT5 ameliorated cardiac hypertrophy by decreasing the expressions of hypertrophic markers and prevented the increase in cell size (Figure 2). In contrast, knockdown of endogenous SIRT5 by RNA interference aggravated cardiac hypertrophy (Figure 3), providing evidence that SIRT5 plays a protective role in the pathological process of myocardial hypertrophy. 

RIP140, a known deleterious regulator of cardiac mitochondrial function, was demonstrated to induce cardiac fibrosis and cardiac hypertrophy (20, 21). Our previous study reported that RIP140 was up-regulated in heart failure and could accelerate the transition from compensated cardiac hypertrophy to heart failure in response to MI stress through repression of mitochondrial function (18, 19). However, the mechanism of RIP140 in regulating cardiac energy homeostasis remains to be elucidated. Our previous research has uncovered that RIP140 and PGC- 1α exert antagonistic roles in regulating cardiac energy state and share many downstream targets, like transcription factors related to fatty acid metabolism (ERRα, PPAR α, PPAR β, and NRF1), as well as their target genes(18, 19, 35, 36). As SIRT5 can be induced by PGC- 1α, it is tempting to speculate that SIRT5 might participate in the cardiac energy homeostasis in the progression of cardiac hypertrophy. In this study, we found that SIRT5 could be negatively regulated by RIP140 (Figure 4) and overexpression of SIRT5 was capable of reversing RIP140-induced cardiac hypertrophy (Figure 5), suggesting that the functional antagonism of RIP140 and PGC-1α in cardiac energy metabolism is possibly associated with the regulation of SIRT5. Indeed, overexpression of SIRT5 is beneficial for mitochondrial function and substance metabolism homeostasis, resembling the effect of PGC-1α but reversing RIP140-mediated metabolic dysregulation in cardiomyocytes (Figure 6). Taken together, these observations further confirm that RIP140 and PGC-1α exert the antagonistic role in maintaining cardiac mitochondrial function and energy homeostasis through oppositely regulating the expression of SIRT5.

Consistent with our previous finding that the deleterious effects of RIP140 under MI stress were associated with the repression of metabolic gene expression, mitochondrial respiration rate, and ATP content (18), the present study also demonstrated that RIP140 induced mitochondrial dysfunction accompanied by the declined expression of Cyt b, ND1 and mt-col, which can be reversed by SIRT5 overexpression (Figure 6A). Additionally, SIRT5 overexpression also improved the functional state of mitochondrial respiration, which was evidenced by the amelioration of the decline in mitochondrial membrane potential and capacity to produce energy induced by RIP140 (Figure 6B-D). However, knocking down SIRT5 based on RIP140 overexpression could only slightly exacerbate RIP140-mediated mitochondrial metabolic disorders, which may be related to the compensatory regulation of energy metabolism in cells. The homeostasis of energy metabolism is upheld through a delicate balance between energy production and consumption, and excessive energy consumption or inhibition may trigger cellular compensatory pathways as protective mechanisms. 

**Table 1:** Detailed information of the materials

Gene/Gene ID	Location	Primer sequences	Base pair length	Amplified product length
SIRT5 (306840)	Chromosome17, NC_086035.1 (21515982..21543529, complement)	Forward: 5'-AACGCAAAGCACATAGTCAT-3' Reverse: 5'-AAGCAAAGGCCAGAGGAGT-3'	20 19	133
RIP140 (304157)	Chromosome11, NC_086029.1 (28382835..28466483, complement)	Forward: 5'-GCCACAGTCAAGCAAACTGG-3' Reverse: 5'-AGGAACACCGCACATTGGAT-3'	20 20	73
Cyt b (26192)	ChromosomeMT, NC_001665.2 (14136..15278)	Forward : 5'-GCAGCTTAACATTCCGCCCAATCA-3' Reverse: 5'-TGTTCTACTGGTTGGCCTCCGATT-3'	24 24	97
ND-1 (26193)	ChromosomeMT, NC_001665.2 (2740..3694)	Forward : 5'-AAGCGGCTCCTTCTCCCTACAAAT-3' Reverse: 5'-GAAGGGAGCTCGATTTGTTTCTGC-3'	24 24	127
mt-co1 (26195)	ChromosomeMT, NC_001665.2 (5323..6867)	Forward : 5'-AAGGTTTGGTCCTGGCCTTA-3' Reverse: 5'-GCAAGGCGTCTTGAGCTAT-3'	20 19	142
β-actin (81822)	Chromosome12, NC_086030.1 (16776664..16779634)	Forward: 5'-TCGTGCGTGACATTAAAGAG-3' Reverse: 5'-ATTGCCGATAGTGATGACCT-3'	20 20	134

DMEM: Dulbecco’s modified eagle medium; TMRE: Tetramethylrhodamine ethyl ester

**Table 2:** Detailed information of primers used for qRT-PCR

Antibody	Application	Company and Catalog NO.
Anti-SIRT5 antibody (Rb)	WB	Abcam (ab259967)
Anti-RIP140 antibody (Rb)	WB	Abcam (ab42126)
Anti-ANF antibody (Rb)	WB	**Santa cruz (sc-20158)**
Anti-BNP antibody (Rb)	WB	**ABclonal (A23996)**
Anti-β-MHC antibody (Rb)	WB	**ABclonal (A7564)**
Anti-α-tubulin antibody (Ms)	WB	Sigma-Aldrich(T6074)
HRP-conjugated secondary antibodies (Rb)	WB	Cell Signaling Technology (7074)
HRP-conjugated secondary antibodies (Ms)	WB	Cell Signaling Technology (7076)

**Table 3:** Detailed information of the antibodies used for Western blot

Kits and Materials	Company and Catalog NO.
ATP Assay Kit	Beyotime (S0026)
BCA Protein Assay Kit	Thermo Scientific (23227)
RNA Reverse Transcription Kit	Thermo Scientific (K1691)
Adeno-X Maxi purification kit	Clontech (631532)
DMEM	Gibco (C11995500BT)
Fetal bovine serum	Gibco (16397-044)
4’, 6-diamidino-2-phenylindole (DAPI)	Invitrogen (P36931)
Penicillin/streptomycin	Invitrogen (15070063)
Lipofectamine 2000	Invitrogen (11668)
Angiotensin Ⅱ(Ang II)	Sigma-Aldrich (4474-91-3)
5-bromodeoxyuridine (BrdU)	Sigma-Aldrich (b5002)
RIPA Lysis Buffer	Beyotime (P0013B)
Trizol	Invitrogen (15596-026)
SYBR qPCR Mix	Thermo Scientific (K0251)
Rhodamine-phalloidin	Cytoskeleton (PHDR1)
TMRE	Sigma-Aldrich (115532-52-0)
MitoXpress ® Xtra reagent	Luxcel Biosciences (MX-400)
SuperSignal^TM^ West Pico PLUS chemical luminescent substrate	Thermo Scientific (34580)

WB: Western Blot; Rb: Rabbit; Ms: Mouse

A. Western blot analysis showed the protein expression of hypertrophyic biomarker ANF in primary NRCMs treated with 100 nM of AngⅡ for indicated time. n=3. B,C. The protein expression of SIRT5 in NRCMs treated with indicated concentrations of AngⅡ for 24 hr or with 100 nM of AngⅡ for indicated time. n=3. D. The SD rats were treated with equal normal saline (NS) or AngⅡ (2 mg/kg/d) for 2 weeks to induce animal model of cardiac hypertrophy. The representative echocardiographic graphs**、**HW/BW ratio and echocardiography data in two groups were shown respectively. E. The protein expression of SIRT5, ANF, and BNP in rat myocardial tissue were detected by Western blot analysis. n=5. Data were shown as mean±SEM. Statistical significance was assessed by one-way analysis of variance (ANOVA) with Bonferroni *post hoc* test for (A-C) and Student’s t-test for (D, E). *P<0.05, **P<0.01 as compared to the Control group

Cardiomyocytes were transfected with plasmid (2000 ng/ml) of pcDNA 3.1(Vector) or SIRT5. A, B. The mRNA level and protein expression of SIRT5 were detected by Quantitative real-time PCR and Western blot, respectively. Cardiomyocytes were treated with Ang Ⅱ (100 nM), simultaneously transfected with SIRT5. C.Western blot analysis showed the protein expression of ANF, β-MHC and SIRT5. D. Cardiomyocytes were dyed with Rhodamine-phalloidin and cell surface areas were captured and measured by High Content Screening system (Scale bar=20 μm). Data were shown as mean±SEM. Statistical significance was assessed by one-way analysis of variance (ANOVA) with Bonferroni post hoc test for (A, C, D). *P<0.05, **P<0.01, ***P<0.001 as compared to the Control group; #*P<*0.05, ^##^*P<*0.01 as compared to the AngII group. n=3

Cardiomyocytes were transfected with three individual siRNA of SIRT5 (marked as si001, si002 and si003) or negative control (NC). A,B. The mRNA level and protein expression of SIRT5 were detected by Quantitative real-time PCR and Western blot, respectively. Primary NRCMs were transfected with siRNA (100pmol/ml) of SIRT5 or Negative Control (NC) for 48 hr. C. Western blot analysis showed that the protein expressions of ANF and β-MHC were increased in si-SIRT5 group. D. Cardiomyocytes were dyed with Rhodamine-phalloidin and cell surface areas were captured and measured by High Content Screening system (Scale bar=20 μm). Data were shown as mean±SEM. Statistical significance was assessed by one-way analysis of variance (ANOVA) with Bonferroni *post hoc* test for (A) and Student’s t-test for (C, D). *P<0.05, **P<0.01 as compared to the NC group. n=3

A. The mRNA level of SIRT5 in primary NRCMs transfected with RIP140 or GFP adenoviruses (MOI 60) for 36 hr was measured by quantitative RT-PCR. B. The protein expression of SIRT5 in Ad-RIP140 cardiomyocytes (MOI 60) for 48 hr was showed by Western blot analysis. C. The mRNA level of SIRT5 was detected by quantitative RT-PCR after cardiomyocytes transfected with siRNA (100 pmol/ml) of negative control (NC) or RIP140 for 48 hr. D. The protein expression of RIP140 and SIRT5 in NC or si-RIP140 cardiomyocytes (100 pmol/ml) for 72 hr was showed by Western blot analysis. Data were shown as mean±SEM. Statistical significance was assessed by one-way analysis of variance (ANOVA) with Bonferroni *post hoc* test for (A, B) and Student’s t-test for (C, D). *P<0.05, **P<0.01 as compared to the Control or NC group. n=3

Cardiomycytes were infected with Ad-RIP140 Adenovirus (MOI 60), simultaneously transfected with SIRT5 plasmid (2000 ng/ml) or siSIRT5 (100 pmol/ml). A. Western blot analysis showed the protein expression of β-MHC, RIP140 and SIRT5. B. Cardiomyocytes were dyed with Rhodamine-phalloidin and cell surface areas were captured and measured by High Content Screening system (Scale bar=20 μm). Data were shown as mean±SEM. Statistical significance was assessed by one-way analysis of variance (ANOVA) with Bonferroni *post hoc* test for (A, B). *P<0.05, **P<0.01 as compared to the Ad-GFP group; #*P<*0.05 as compared to the Ad-RIP140 group. n=3

Cardiomyocytes were transfected with Ad-GFP or Ad-RIP140 (MOI 60, 42 hr), following treatment with SIRT5 plasmid (2000 ng/ml, 48h) or siRNA (100 pmol/ml, 48 hr). A. Quantitative real-time PCR results of mitochondrial DNA versus nuclear DNA ratios. B. TMRE fluorescence analysis was used for detecting the mitochondrial membrane potential (Scale bar=20 μm). C. Relative Fluorescence Unit (RFU) in cardiomyocytes at real time to estimate the relative oxygen consumption. D. Relative ATP content in NRCMs was examined by ATP Assay Kit. Data were shown as mean±SEM. Statistical significance was assessed by one-way analysis of variance (ANOVA) with Bonferroni *post hoc* test for (A-D). *P<0.05, **P<0.01, ***P<0.001 as compared to the Ad-GFP group. #*P<*0.05, ^##^*P<*0.01, ^###^*P<*0.001 as compared to the Ad-RIP140 group. n=3

## Conclusion

In summary, this study unveils a novel mechanism whereby SIRT5 emerges as a crucial regulator in maintaining mitochondrial homeostasis and ultimately safeguarding against the progression of cardiac hypertrophy under the regulation of RIP140. These findings offer fresh insights into the role of RIP140 in governing cardiac energy metabolism and highlight the RIP140-SIRT5 axis as a promising therapeutic target for the treatment of cardiac hypertrophy. 

## Declaration

We have not used any AI tools or technologies to prepare this manuscript.
